# G-Quadruplex DNA Sequences Are Evolutionarily Conserved and Associated with Distinct Genomic Features in *Saccharomyces cerevisiae*

**DOI:** 10.1371/journal.pcbi.1000861

**Published:** 2010-07-22

**Authors:** John A. Capra, Katrin Paeschke, Mona Singh, Virginia A. Zakian

**Affiliations:** 1 Department of Computer Science, Lewis-Sigler Institute for Integrative Genomics, Princeton University, Princeton, New Jersey, United States of America; 2 Department of Molecular Biology, Princeton University, Princeton, New Jersey, United States of America; Washington University School of Medicine, United States of America

## Abstract

G-quadruplex DNA is a four-stranded DNA structure formed by non-Watson-Crick base pairing between stacked sets of four guanines. Many possible functions have been proposed for this structure, but its *in vivo* role in the cell is still largely unresolved. We carried out a genome-wide survey of the evolutionary conservation of regions with the potential to form G-quadruplex DNA structures (G4 DNA motifs) across seven yeast species. We found that G4 DNA motifs were significantly more conserved than expected by chance, and the nucleotide-level conservation patterns suggested that the motif conservation was the result of the formation of G4 DNA structures. We characterized the association of conserved and non-conserved G4 DNA motifs in *Saccharomyces cerevisiae* with more than 40 known genome features and gene classes. Our comprehensive, integrated evolutionary and functional analysis confirmed the previously observed associations of G4 DNA motifs with promoter regions and the rDNA, and it identified several previously unrecognized associations of G4 DNA motifs with genomic features, such as mitotic and meiotic double-strand break sites (DSBs). Conserved G4 DNA motifs maintained strong associations with promoters and the rDNA, but not with DSBs. We also performed the first analysis of G4 DNA motifs in the mitochondria, and surprisingly found a tenfold higher concentration of the motifs in the AT-rich yeast mitochondrial DNA than in nuclear DNA. The evolutionary conservation of the G4 DNA motif and its association with specific genome features supports the hypothesis that G4 DNA has *in vivo* functions that are under evolutionary constraint.

## Author Summary

DNA can form structures other than the traditional double helix. The G-quadruplex, a stable four-stranded structure formed by guanine-rich DNA, is one such alternative structure. Sequence motifs with the potential to form G-quadruplex structures (G4 DNA motifs) are found in the genomes of many species. However, since such motifs can occur by chance, it is not known which sequence regions form G-quadruplexes *in vivo*, and if formed, how they function. Evolutionary conservation of a sequence across species provides evidence of a biologically important function. We found that G4 DNA motifs in *S. cerevisiae* are more conserved than expected among related fungi, and the patterns of this conservation suggest that many of the motifs form functional G-quadruplexes. We explored potential functions of the G-quadruplex by identifying significant associations of motifs with annotated genome features. Our analysis corroborated and refined previous hypotheses about the importance of G-quadruplexes in telomeres and gene promoters, and suggested intriguing additional roles in double strand break processing and the mitochondria.

## Introduction

**Figure 1:**
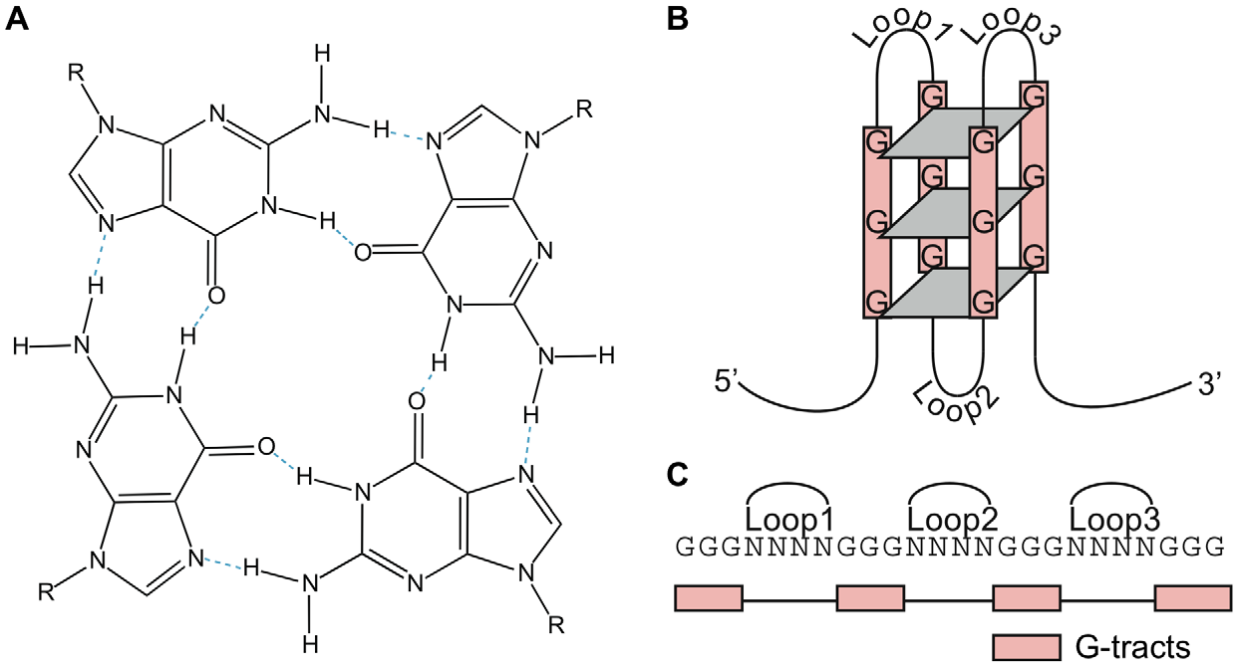
The G4 DNA structure and motif. (**A**) Structure of a G-quartet. The planar ring of four hydrogen-bonded guanines is formed by guanines from different G-tracts, which are separated by intervening loop regions in the intra-molecular G4 DNA structure. (**B**) Schematic of an intra-molecular G4 DNA structure consisting of three G-quartets. Inter-molecular G4 DNA structures can also form from two or four strands. (**C**) The G4 DNA motif sequence used in this study with four G-tracts of three guanines separated by loop regions.

DNA primarily exists as a double helix. However, DNA can also adopt other structural conformations that have the potential to play critical roles in a range of biological processes. One such structure is G-quadruplex DNA (G4 DNA structure), which was discovered in the late 1980s when biochemical experiments demonstrated that oligodeoxynucleotides that contain four separated runs of two, three, or four guanines (G-tracts) can spontaneously form four-stranded structures [1], [2] (Fig. 1A). G4 DNA structures consist of stacked planar G-quartets that are held together by Hoogsteen hydrogen bonding between four guanines from each of the G-tracts (Fig. 1B). The guanines can come from a single nucleic acid strand (intra-molecular) or multiple strands (inter-molecular), and the strands may be oriented in a parallel or anti-parallel orientation. G4 DNA structures are compact, highly stable under physiological pH and salt conditions, resistant to degradation by nucleases, and can have melting temperatures even higher than that of duplex DNA [3], [4]. G4 DNA structures can be formed from runs of two guanines, but they are less stable than those with longer runs.

The G4 DNA structure is of considerable interest because of its potential to influence a variety of biological processes [3], [5]. For example, telomeric DNA in most eukaryotic organisms consists of G-rich repeated sequence ending with a 3′ single stranded G-rich overhang that can form G-quadruplexes *in vitro* [1], [2]. The first direct evidence for the presence of G4 DNA structures *in vivo* came from studies using G4 DNA-specific antibodies to detect intermolecular structures at ciliate telomeres where their formation and dissolution are cell cycle regulated [6]-[8]. However, as described in detail in this paper, telomeric DNAs are not the only chromosomal sequences with the ability to form G4 DNA structures.

Because experimental characterization of the *in vivo* functions of G4 DNA structures has proved difficult [9], especially at non-telomeric loci, genome-wide computational analyses have played an increasing role in the identification of regions that have the potential to form G4 DNA structures (G4 DNA motifs). The distribution of G4 DNA motifs has been investigated in *S. cerevisiae* [10], human [11], [12], and a number of prokaryotic genomes [13] in the hope that the patterns of occurrence will provide insight into the functional roles of these structures. In each case, a computational search for variations of the G4 DNA motif, usually four tracts of three or more guanines separated by loop regions of any nucleotide, was performed (Fig. 1C). Across a wide range of species, G4 DNA motifs were found in telomeres, G-rich micro- and mini-satellites, near promoters, and within the ribosomal DNA (rDNA) [14]–[16]. In the human genome, genes that are near G4 DNA motifs fall into specific functional classes; for example, oncogenes and tumor suppressor genes have particularly high or low G4 DNA forming potential [17]–[19]. Recently, human G4 DNA motifs were reported be associated with recombination prone regions [20] and to show mutational patterns that preserved the potential to form G4 DNA structures [16]. Computational analysis in *S. cerevisiae* identified several hundred G4 DNA motifs, and found them to be significantly associated with promoter regions and to a lesser extent with open reading frames (ORFs) [10]. Thus, studies in a wide range of organisms have led to the proposal that G4 DNA structures affect multiple cellular processes beyond their roles at telomeres. However, direct support for formation and function of G4 DNA structures *in vivo* is still largely unavailable.

In this study, we integrated genome sequence data, experimental analysis, and computational exploration of genome annotations to investigate the conservation and function of G4 DNA structures in *S. cerevisiae*. Evolutionary conservation across related species has played a vital role in defining functional elements such as genes and regulatory sites [21], [22]. We identified sequence motifs with the potential to form G-quadruplex structures in *S. cerevisiae* and six other fungal species and assessed the evolutionary sequence conservation of the motifs across these seven species. We found that G4 DNA motifs and the nucleotides comprising them were more evolutionarily conserved than expected by chance; however, they were not as strongly conserved as genes and many known regulatory sites. Additionally, the patterns of nucleotide conservation within the motifs indicated that the evolutionary constraint was likely the result of pressure to maintain the ability of these motifs to form G4 DNA structures. This analysis provides strong evidence that many computationally identified G4 DNA motifs form functional G4 DNA structures *in vivo*. To characterize possible functions for the structures, we evaluated the association of conserved and non-conserved G4 DNA motifs with a range of genomic features. These tests corroborated previous observations of the significant associations of G4 DNA motifs with gene promoters and rDNA [10], and suggested several new potential biological functions, such as roles in double strand break repair and in the mitochondrial genome.

## Results

### Sequence conservation of G4 DNA motifs

#### Preliminaries

The genome-wide occurrence of sequence motifs with the potential to form G-quadruplex DNA in *S. cerevisiae* was identified previously [10], but the presence of a predicted motif in a DNA sequence does not imply that it forms a G4 DNA structure *in vivo*. Sequences with the potential to form G4 DNA structures occur frequently by chance [11]. The conservation of a motif across related species provides evidence that the motif is under evolutionary constraint and suggests that it serves some function.

To characterize the conservation of G4 DNA motifs in the *S. cerevisiae* genome [23] across closely related species, we defined a set of candidate DNA regions using an algorithm similar to those used previously [10]–[14], [24], [25]. We sought DNA regions with at least four tracts of three or more consecutive guanines (G-tracts) separated by loop regions of limited length (Fig. 1C). We considered loop length thresholds in the range of 5 to 50 nucleotides (nt). Unless otherwise stated, all results are based on the set of motifs with loops of 25 nt or less. (The genome locations of these G4 DNA motifs are given in Dataset S1.) This threshold was selected to include the longest previously reported loop in a G4 DNA structure (22 nt) [26]. Regions containing more than four G-tracts separated by less than a given loop length threshold were counted as a single motif. (See Methods for a comparison of our motif definition with previous approaches.) In this paper, “G4 DNA motif” refers to DNA sites that match this sequence pattern, and “G4 DNA structure” refers to the G-quadruplex DNA secondary structure potentially formed by sequences containing the G4 DNA motif.

**Figure 2:**
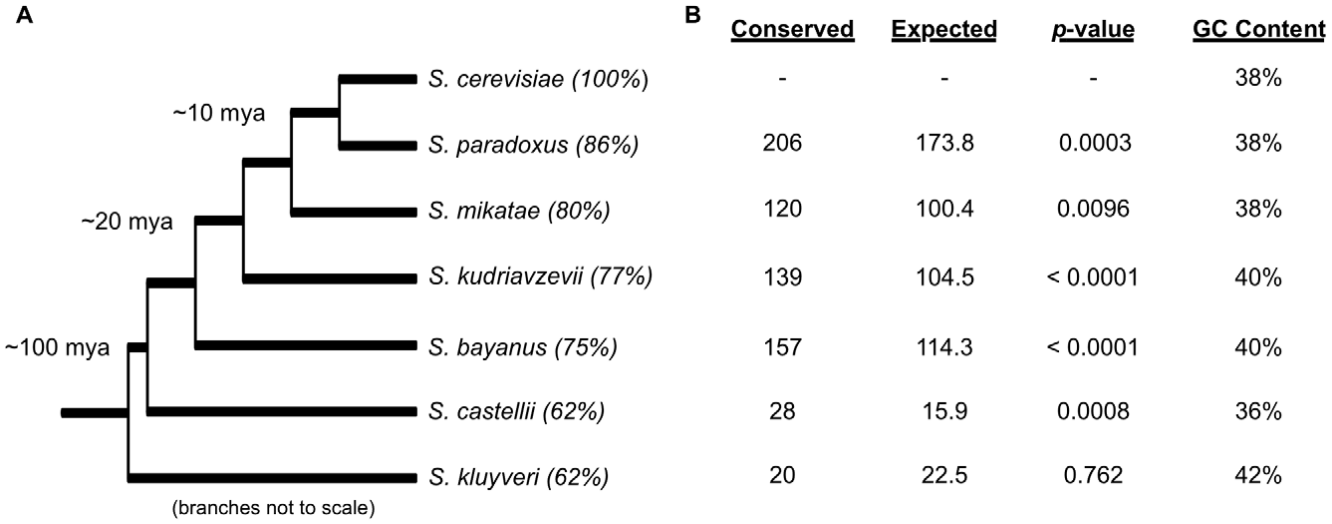
The evolutionary conservation of G4 DNA motifs between *S. cerevisiae* and six related yeast species. (**A**) The phylogenetic tree for the seven yeast species considered in this study (not to scale). The four *sensu stricto* species (*S. paradoxus*, *S. mikatae*, *S. kudriavzevii*, *S. bayanus*) diverged from *S. cerevisiae* within the last ∼20 million years. *S. castelli* and *S. kluyveri* are considerably more distant [21]. The percent sequence identity to *S. cerevisiae* over the alignable regions is given in parentheses. (**B**) The evolutionary conservation of the 507 non-telomeric, nuclear *S. cerevisiae* G4 DNA motifs in sequence regions that could be aligned to at least one other genome. Significantly more G4 DNA motifs were conserved than expected by chance between *S. cerevisiae* and five of the six species considered. The one exception was *S. kluyveri*, which is the most distant and GC-rich species among the seven yeasts.

We analyzed the sequence conservation of *S. cerevisiae* G4 DNA motifs across the genomes of seven *Saccharomyces* strains (Fig. 2A). The five *sensu stricto* strains (*S. cerevisiae*, *S. paradoxus*, *S. mikatae*, *S. kudriavzevii*, *S. bayanus*) are estimated to have diverged from each other ∼10–20 million years ago, while *S. castellii* and *S. kluyveri* are more evolutionarily distant from *S. cerevisiae* (∼100 million years since divergence) [21]. The overall number of G4 DNA motifs found in each of these species was within a factor of two of the number found in *S. cerevisiae* (Table 1).

**Table 1:** Number of G4 DNA motifs found in each species.

Species	G4 DNA Motifs	Number of Nucleotides Considered (kb)	G4 DNA motif density (motif/kb)
*S. cerevisiae*	668	12156	0.055
*S. paradoxus*	731	11873	0.062
*S. mikatae*	567	11470	0.049
*S. kudriavzevii*	940	11132	0.084
*S. bayanus*	1298	11478	0.113
*S. castelli*	869	11242	0.077
*S. kluyveri*	1258	10991	0.114

The number of G4 DNA motifs in each species is based on all available sequence data; however, the quality and coverage of the sequences available for each genome varies.

Due to lack of available sequence data and the difficulty of aligning repeat-rich regions from telomeric and mitochondrial DNAs, G4 DNA motifs in these regions were not included in the conservation analysis. In addition, we only considered motifs found in one complete copy of the rDNA repeat. After these filtering steps, 552 of the 668 total *S. cerevisiae* G4 DNA motifs we identified remained, and 507 of these were in regions that could be aligned to at least one other species.

**Figure 3:**
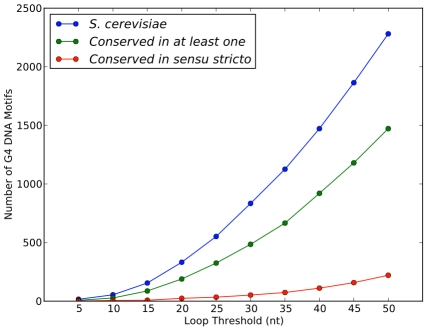
The conservation of G4 DNA motifs as a function of the loop length threshold. The number of G4 DNA motifs identified in *S. cerevisiae* increases as maximum loop length limit is increased (blue line). More than half of the motifs are conserved in at least one other species at each loop threshold (green line). The number of motifs conserved in all *sensu stricto* species also increases as longer loops are tolerated (red line).

The number of non-telomeric, nuclear G4 DNA motifs found in the *S. cerevisiae* genome increased with the maximum length allowed for the loop regions (Fig. 3). For example, there were 54 motifs with all loops less than or equal to 10 nt, and 552 G4 DNA motifs with loops less than or equal to 25 nt. This increasing pattern is similar to that observed in a previous analysis [10] that placed an explicit limit on motif length and did not constrain the loops.

#### *S. cerevisiae* G4 DNA motifs are more conserved across related species than expected by chance

We evaluated the conservation of the G4 DNA motifs on both the motif-level and nucleotide level. In the motif analysis, we determined if G4 DNA motifs occurred at the corresponding location in the genomes of *S. cerevisiae* and each of the other species considered. For a *S. cerevisiae* motif to be considered conserved in another species, a G4 DNA motif had to be present in a sequence position that overlapped that of the *S. cerevisiae* motif. We did not require conservation of the exact sequence of the motif; that is, conserved motifs could have different loop sizes, number of G-tracts, and nucleotide sequences.

The majority of the *S. cerevisiae* G4 DNA motifs included in the evolutionary analysis were conserved in at least one other species (Fig. 3). This result held for loop length thresholds from 5 to 50 nt. The number of motifs conserved across all *sensu stricto* yeasts also increased with the maximum allowed loop length, but compared to shorter G4 DNA motifs, a smaller fraction of the long motifs exhibited this deep conservation. At the 25 nt loop length threshold, 64% (324 of 507) of the alignable G4 DNA motifs were conserved in at least one other species, and 7% (34 of 507) were conserved across all of the *sensu stricto* species. The latter group will be referred to as the “conserved” G4 DNA motifs. (The genome locations of all conserved G4 DNA motifs are given in Dataset S2.)

The number of G4 DNA motifs conserved between *S. cerevisiae* and each of the six other related fungi is given in Fig. 2B. As expected, the number of conserved motifs roughly followed the evolutionary distances between the species: each of the *sensu stricto* species had considerably more conserved motifs than the two more distantly related species (*S. castellii*, *S. kluyveri*).

Significantly more G4 DNA motifs were conserved than expected between *S. cerevisiae* and five of the six related species (see Fig. 2B for p-values). For example, 206 motifs were conserved between *S. cerevisiae* and *S. paradoxus*. The expected number of motifs conserved between *S. cerevisiae* and a genome with the sequence content and evolutionary distance of *S. paradoxus* is 174. The expected number of conserved motifs was estimated by generating 10,000 random genomes and their corresponding alignments to *S. cerevisiae* using an evolutionary model that maintained the substitution probabilities and evolutionary distance between each species pair, but had no explicit pressure to maintain the motifs (see Methods). Only three out of the 10,000 random genomes yielded as many conserved motifs as were observed. However, the *S. cerevisiae* G4 DNA motifs were not more conserved than expected with *S. kluyveri*, the most distant and most GC-rich species considered. The greater GC content of this species made it more likely that a G4 DNA motif would be conserved by chance than in species with a GC content similar to that of *S. cerevisiae*.

#### Nucleotide conservation patterns in G4 DNA motifs indicate pressure to maintain the G4 DNA structure

The above results suggest that G4 DNA motifs are more conserved across species than expected. However, these findings do not explicitly tie this conservation to the formation of the G4 DNA structure. It is possible that the motifs are conserved due to associations with other genomic features that are responsible for the observed evolutionary conservation. These possibilities can be distinguished by considering G4 DNA motif conservation at the nucleotide level.

In the nucleotide level analysis, each individual nucleotide position in the sequence alignments was assigned a value that quantified its evolutionary conservation. We compared the evolutionary conservation of different sets of nucleotides from *S. cerevisiae* G4 DNA motifs and the surrounding sequence regions. The nucleotide conservation scores were taken from the phastCons program, which assigns each alignment column a value between zero and one that represents the posterior probability that the position is conserved according to a phylogenetic Hidden Markov Model [27], [28].

The nucleotides of 308 of the 507 alignable G4 DNA motifs (61%) were more conserved than the 100 nucleotides on either side of the motif. This conservation was significantly greater than that expected by chance (p = 7.394×10^−7^, binomial test). In a similar test that pooled nucleotides across G4 DNA motifs, we found that the average conservation of all the nucleotides over all of the motifs was significantly higher than the average conservation of all positions in neighboring regions within 100 nt on either side (p<2.2×10^−16^ for difference, Wilcoxon rank sum test) (Table 2). This p-value was so low because there were thousands of nucleotides representing the two groups being compared. In both of these tests, nucleotides in G4 DNA motifs were significantly more conserved than neighboring nucleotides. In addition, the motif nucleotides were also significantly more conserved (p<2.2×10^−16^, Wilcoxon rank sum test) than the average conservation of all nucleotides in the alignments.

**Table 2:** Conservation of G4 DNA Motif Nucleotides.

Comparison	Nucleotide sets	Avg. Conservation	p-value
Neighborhood	G4 DNA motifs	**0.665**	<2.2×10^−16^
	Neighboring 200 nt	0.646	
Within Motif	Disruptive Positions	**0.670**	2.365×10^−9^
	Non-disruptive Positions	0.631	

This table compares the average conservation of different sets of nucleotides in and around G4 DNA motifs. The first comparison demonstrates that nucleotides in G4 DNA motifs are significantly more conserved (bold) than those in the surrounding sequence positions (*p*<2.2×10^−16^). The second comparison demonstrates that within G-tracts of G4 DNA motifs, disruptive positions are significantly more conserved than non-disruptive positions (*p* = 2.365×10^−9^).

Analyzing nucleotide conservation patterns also allowed us to demonstrate that the conservation of the motifs is likely due to evolutionary pressure to maintain the ability to form G4 DNA structures. The G4 DNA motif consists of four tracts of at least three guanines separated by short loop regions that can contain any nucleotide (Fig. 1C). If a motif forms a functional G4 DNA structure, we would expect mutations that disrupt G-tracts to be more rare than those that maintain G-tracts, since the disruption of a G-tract prevents the formation of the G4 DNA structure [16]. Thus, we compared the conservation of disruptive and non-disruptive nucleotide positions within G-tracts. In the motif, a minimum of three guanines in a G-tract is required for stable G4 DNA structure formation, but many G-tracts have more than three guanines. For example, in a G-tract of four G's (GGGG), a mutation of the first or last guanine (non-disruptive positions) would not break the G4 DNA motif pattern while a mutation in either of the two middle positions (disruptive positions) would prevent formation. We compared these two types of G-tract nucleotides in *S. cerevisiae*, and found that disruptive positions were significantly more conserved than non-disruptive positions (0.670 v. 0.631, p = 2.365×10^−9^, Wilcoxon rank sum test) (Table 2).

The motif-level conserved G4 DNA motifs exhibited similar nucleotide conservation patterns. The average conservation of conserved motif nucleotides was significantly greater than that of the surrounding nucleotides (0.722 v. 0.652, p<2.2×10^−16^). The disruptive positions showed an even greater difference in nucleotide conservation with the non-disruptive positions (0.778 v. 0.641, p = 1.987×10^−6^).

### The distribution of conserved and non-conserved G4 DNA motifs in the *S. cerevisiae* genome

**Figure 4:**
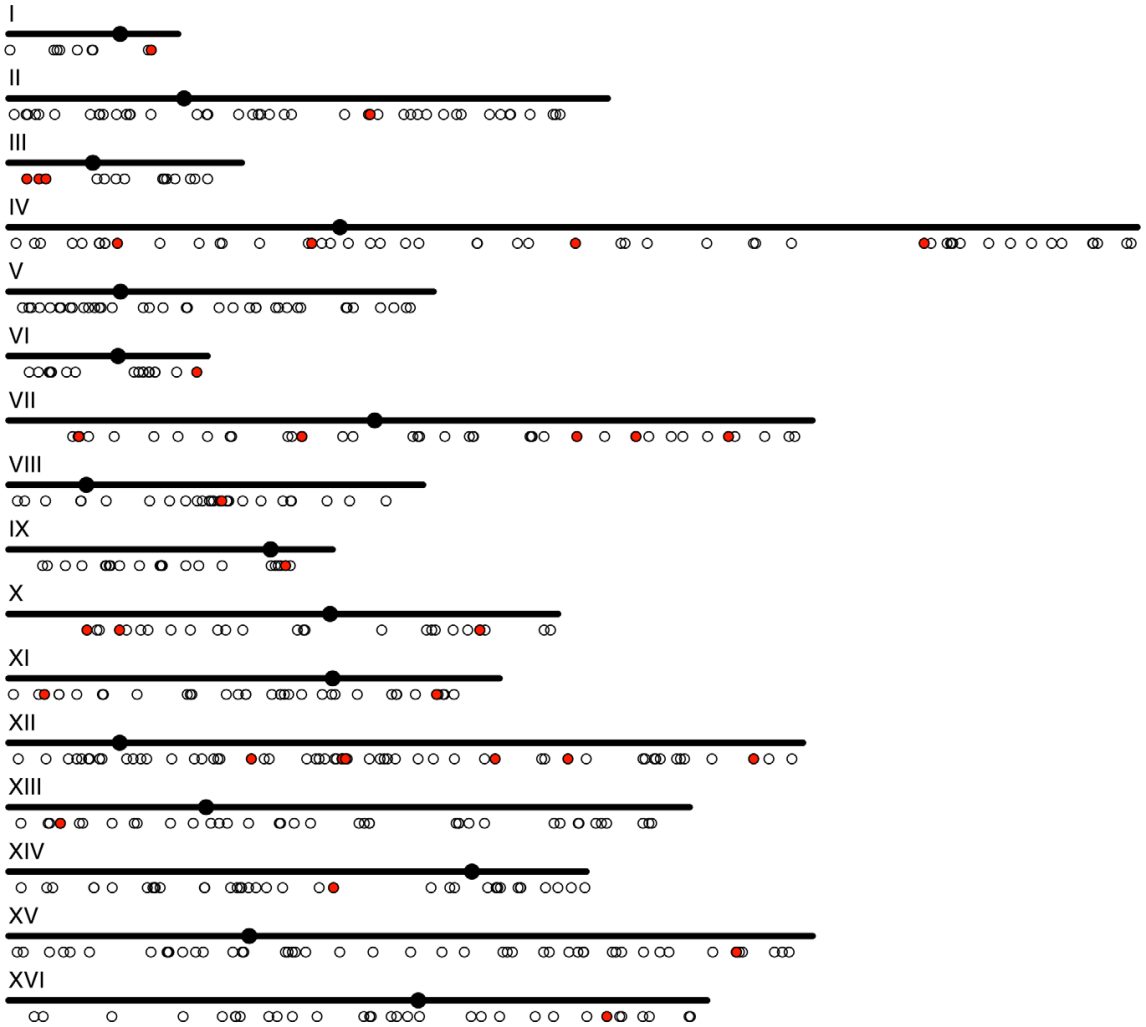
The distribution of G4 DNA motifs across the *S. cerevisiae* nuclear genome. Each small circle represents the location of a G4 DNA motif in a chromosome. Motifs conserved across the *sensu stricto* species are highlighted in red.

Disregarding the G-rich telomeres, the distribution of G4 DNA motifs across the 16 yeast nuclear chromosomes was relatively constant (Fig. 4). Chromosome XVI had the lowest density of G4 DNA motifs with 0.034 G4 sites per kb of sequence, while chromosome VI had the highest at 0.067 per kb. G4 DNA motifs were found across the entire length of most chromosomes (Fig. 4). The longest stretch of the genome without a G4 DNA motif was a 180 kb ORF-rich region on the right arm of chromosome IV (1062535–1242537). A region on the right arm of chromosome VI had the highest density of G4 DNA with seven G4 DNA motifs within ∼16 kb (182580–199009). This region contains a variety of functional elements, including an autonomously replicated sequence (ARS), tRNA genes, several ORFs, and a Ty1 retrotransposon.

The G4 DNA motifs conserved in all of the *sensu stricto* species were also distributed across the genome. At least one was found on each chromosome, except for chromosome V (Fig. 4). These motifs occurred in a range of genomic contexts; 11 out of 34 were found in ORFs. This is significantly fewer than expected by chance (q<0.0001). Four motifs were conserved across all seven species. Two of these were in the coding regions for the mature ribosomal DNA, and two were within the ORFs of conserved genes (MEP3, an ammonium transporter; PBP1, a protein involved in response to glucose deprivation). Since each of these conserved G4 DNA motifs was in a region likely constrained for other functional reasons, we cannot attribute their conservation to their ability to form G4 DNA structures.

### The length and physical properties of conserved and non-conserved G4 DNA motifs

Conserved G4 DNA motifs were on average longer and had more G-tracts than non-conserved motifs (Table 3). Non-telomeric, non-mitochondrial G4 DNA motifs ranged in length from 17 to 162 nt with an average of 54.3±16.8 nt (± standard deviation), while the average conserved motif was 63.3±28.3 nt (p = 0.137, Wilcoxon rank-sum test). When interpreting these statistics, it is important to note that regions with more than four G-tracts in close proximity were counted as a single G4 motif. These sites have the potential to form several different G4 DNA structures; this was found in 50% (17/34) of the conserved motifs but in only 22% (120/552) of all G4 DNA motifs (p = 1.58×10^−4^, Fisher's exact test). Reflecting this pattern, conserved G4 DNA motifs had an average of 5.1±1.9 G-tracts, while all motifs had only 4.3±0.8 (p = 1.47×10^−5^). The G-tracts within conserved G4 DNA motifs were also longer (3.7±1.4 nt) than average (3.3±0.8 nt) (p = 3.65×10^−10^). In contrast, the average loop length was shorter among the conserved motifs (10.8±6.9 nt) than in all motifs (12.2±7.2 nt) (p = 0.0169). G4 DNA structures can vary in the number of stacked G-quartets they contain. This number is determined by the number of consecutive guanines found over the G-tracts of the G4 DNA motif. As expected based on the greater average G-tract length in conserved G4 motifs, the average number of stacked G-quartets is greater for conserved motifs (3.3±0.4) than overall (3.0±0.2) (p<2.2×10^−16^). However, the vast majority of G4 DNA motifs (both conserved and non-conserved) only have the potential to form structures with three stacked G-quartets. Finally, as noted above, disruptive positions in conserved G4 motifs showed a greater difference in nucleotide conservation as compared to non-disruptive positions than did non-conserved G4 motifs.

**Table 3:** Comparison of the length and physical properties of all nuclear, non-telomeric G4 DNA motifs in *S. cerevisiae* with those conserved across the *sensu stricto* species.

Property	All G4 DNA (552)	*Sensu stricto* Conserved (34)
Length	54.3 nt	63.3 nt
# G-tracts	4.3	5.1
# Loops	3.3	4.1
Avg. G-tract Length	3.3 nt	3.7 nt
Avg. Loop Length	12.2 nt	10.8 nt
Avg. # Stacked G-tetrads	3.0	3.3

Conserved G4 DNA motifs are on average longer and have more G-tracts than other G4 DNA motifs. Their individual G-tracts are longer as well. See the text for p-values.

### Associations between G4 DNA motifs and genome features

To investigate potential functional roles of G4 DNA motifs, we computed the association between the motifs (both conserved and non-conserved) and an extensive set of functional genomic features (Table 4, Datasets S3 and S4). An earlier analysis of the *S. cerevisiae* genome computed the enrichment of G4 DNA motifs in yeast promoters and ORFs by generating random sets of promoters and ORFs based on a position-specific transition matrix and found that both classes of sequences were enriched in G4 DNA motifs [10]. Here we greatly extend this earlier analysis by quantifying the association of *S. cerevisiae* G4 DNA motifs with more than 40 genome features and gene classes that includes those curated by the *Saccharomyces* Genome Database (SGD), along with many other experimentally determined genome features that have not yet been annotated in the SGD. (See Methods for the full list.) We developed a new genome-wide method to assess the significance of the association (and lack of association) between G4 DNA motifs and genome features that takes into account their lengths and chromosomal distributions (Methods). To account for the testing of multiple association hypotheses, we control the false discovery rate (FDR) and report a q-value for each test [29], [30]. In this context, q-values are analogous to p-values and can be interpreted as the minimum false positive rate at which an association can be called significant. We use a q-value threshold of 0.05 (an expected false positive rate of 5%).

**Table 4:** Significant associations between genome features and G4 DNA motifs.

		All G4 DNA		Conserved G4 DNA	
Genome Features (window size)	# Features	# motifs	q-value	# motifs	q-value
γ-H2A Binding Site (500)	4123	181	<0.001	9	0.335
Double Promoter (0)	650	87	<0.001	10	<0.001
TF Binding Site (0)	3328	34	<0.001	7	<0.001
Double Strand Break Site (500)	2313	160	<0.001	7	1
Non-essential ORF (500)	4683	464	<0.001	10	1
Dubious ORF (0)	761	28	<0.001	7	0.162
Promoter (0)	5720	308	0.005	21	0.441
rDNA (0)	1	4	0.005	3	<0.001

For each genome feature, the number of instances of that feature in the genome, the number of overlapping G4 DNA motifs, and the significance of this overlap are given. Several of the associations (for example, with double promoters, TF binding sites, and the rDNA) support previous hypotheses about the function of G4 DNA motifs. The significant associations with γ-H2AX and DSB sites and different classes of ORFs suggest additional potential functions for the motif (see Discussion). The conserved G4 DNA motifs maintain the significant associations with TF binding sites, promoters, and the rDNA, but the associations with the other features are no longer significant. For some features, a window of surrounding nucleotides was used to determine associations. In these cases, the size of the window used in the analysis is indicated in parentheses after the feature name.

Multiple genome features were found to be significantly associated with G4 DNA motifs (Table 4). First, we describe the results for the full set of all nuclear, non-telomeric G4 DNA motifs, and then we contrast these with the associations observed for the highly conserved motifs. Several of the significant associations for the full motif set support previous findings [10], [15], [18], [24], [31]; G4 DNA motifs were strongly associated with single and double promoters (q = 0.005, q<0.001) and transcription factor (TF) binding sites [32] (q<0.001). We also found a previously unrecognized association between G4 DNA motifs and regions within 500 nt of non-essential ORFs (q<0.001) that could reflect a function for the G4 DNA structure in regulating this gene class. We also confirmed the previous finding that G4 DNA motifs were significantly associated with the rDNA repeat (q = 0.005). G4 DNA motifs were also significantly more likely to be found in ORFs annotated as “dubious” in the SGD than expected by chance (q<0.001). In contrast, the association with all confirmed ORFs was not statistically significant (q = 1.0). A previous study found that G4 DNA motifs were significantly enriched in *S. cerevisiae* ORFs [10], but it considered dubious and verified ORFs together.

We discovered several associations that may indicate further functions of G4 DNA structures (Table 4). Since G4 DNA structures can form only when the DNA is in a single-stranded state, we analyzed regions of the *S. cerevisiae* genome where the DNA has a propensity to be single-stranded. In particular, we evaluated the association of G4 DNA motifs with sites of spontaneous DNA damage as marked by a high content of phosphorylated H2A (hereafter called γ-H2AX), an early and eukaryotic-wide marker of DNA damage. (Both of the *S. cerevisiae* H2A genes are the equivalent of the H2AX variant in more complex cells; in yeast, H2A is phosphorylated but not replaced in response to DNA damage [33], [34]). The positions of these sites in cells that have not been exposed to DNA damaging agents mark regions of the genome that are particularly susceptible to breakage, which likely occurs mainly as a result of impaired DNA replication. The occurrence of G4 DNA motifs was significantly associated with genome wide sites of high γ-H2AX binding (q<0.001). This finding was originally based on genome wide γ-H2AX binding sites identified using a high-density whole genome array (M. Grunstein, personal communication); however, it also held for sites of high γ-H2AX binding determined using a genome wide ChIP on Chip array (see Methods). The reported q-values are based on our ChIP-chip analysis.

DNA double strand breaks occur much more often in meiotic than in mitotic cells, where they are initated via the action of the Spo11 nuclease and lead to meiotic recombination [35]. Using a genome-wide map of preferred meiotic DSB sites, determined by identifying single-stranded DNA formed at meiotic DSBs [36], we found a significant association between G4 DNA motifs and these experimentally determined meiotic DSB sites (q<0.001).

The 34 G4 DNA motifs conserved across the *sensu stricto* species maintained the significant associations with TF binding sites (q<0.001), double promoters (q<0.001), and the rDNA (q<0.001). However, for these conserved motifs, the associations with DSBs (q = 1.0), γ-H2AX binding sites (q = 0.335), and dubious ORFs (q = 0.162) were not significant. Though these associations did not pass the significance threshold, each of these features was among the strongest associations for conserved motifs (Dataset S4).

In addition, we identified genome features that were significantly under-associated with G4 DNA motifs; that is, they overlapped with G4 DNA motifs significantly less than expected by chance. For all nuclear, non-telomeric G4 DNA motifs, the only significant under-associations were with essential ORFs (q = 0.032) and the first 100 nucleotides upstream of ORFs (q<0.001). The conserved G4 DNA motifs are associated with both ORFs and coding sequences significantly less than expected (q<0.001 for both). This is particularly notable given the high conservation of these regions.

As described above, we observed significant associations between G4 DNA motifs and promoter regions and TF binding sites. G-rich regulatory motifs have been shown to be largely responsible for the enrichment of G4 DNA motifs in regions upstream of transcription start sites in human genes [14], [15], [31]. We explored the effect of known TF binding sites in yeast on the significant association between G4 DNA motifs and promoter regions by examining the overlaps of G4 DNA motifs and the 3328 known TF binding sites as defined in SGD [32]. G4 DNA motifs and TF binding sites were significantly associated (q<0.001). Moreover, the significant association of G4 DNA motifs and promoter regions held when the motifs that directly overlap TF binding sites were not considered (q = 0.001). Thus, this association is likely not due solely to the presence of G-rich TF recognition sites in yeast promoters.

### Mitochondrial G4 DNA motifs

**Figure 5:**
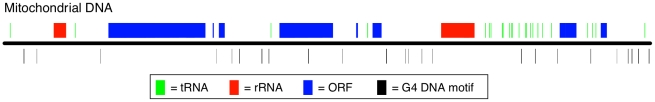
The distribution of G4 DNA motifs across the *S. cerevisiae* mitochondrial DNA. The horizontal black line represents the 75kb mtDNA genome. The rectangles above mark the location of tRNA genes (green), rRNA genes (red), and ORFs (blue). ORFs that encode multiple genes (like COX1, subunit I of cytochrome c oxidase) are drawn as a single rectangle. The vertical black lines below indicate the location of the 32 G4 DNA motifs across the mtDNA. The width of these lines reflects the actual length of the G4 DNA motif. (Note that several motifs are so close that they overlap in the figure.) All motifs are drawn below the mtDNA sequence regardless of the strand on which they occur. The distribution of mitochondiral G4 DNA motifs is biased against overlapping these genomic features; only two of the 32 motifs overlap a tRNA, rRNA, or ORF (*q*<0.001).

A previous analysis of the G4 DNA motifs in the yeast genome did not consider the mtDNA [10]. We found that mtDNA had an order of magnitude more G4 DNA motifs per kb (0.373) than any of the nuclear chromosomes (0.034–0.067). This high density is surprising because the yeast mtDNA is far more AT-rich than the nuclear genome (83% AT in mtDNA compared to 62% AT genome-wide). The distribution of the G4 DNA motifs across the mtDNA was biased towards regions that do not encode ORFs, rRNA, or tRNA genes (Fig. 5). Only two of the 32 mitochondrial G4 DNA motifs overlapped an ORF, and none of the motifs overlapped tRNA or rRNA genes (q<0.001 for this amount of overlap with mitochondrial features). Due to the lack of sequence information for the mtDNA in other fungi, we were not able to analyze the evolutionary conservation of the mitochondrial G4 DNA motifs.

### G4 DNA motifs form G4 DNA structures *in vitro*

**Figure 6:**
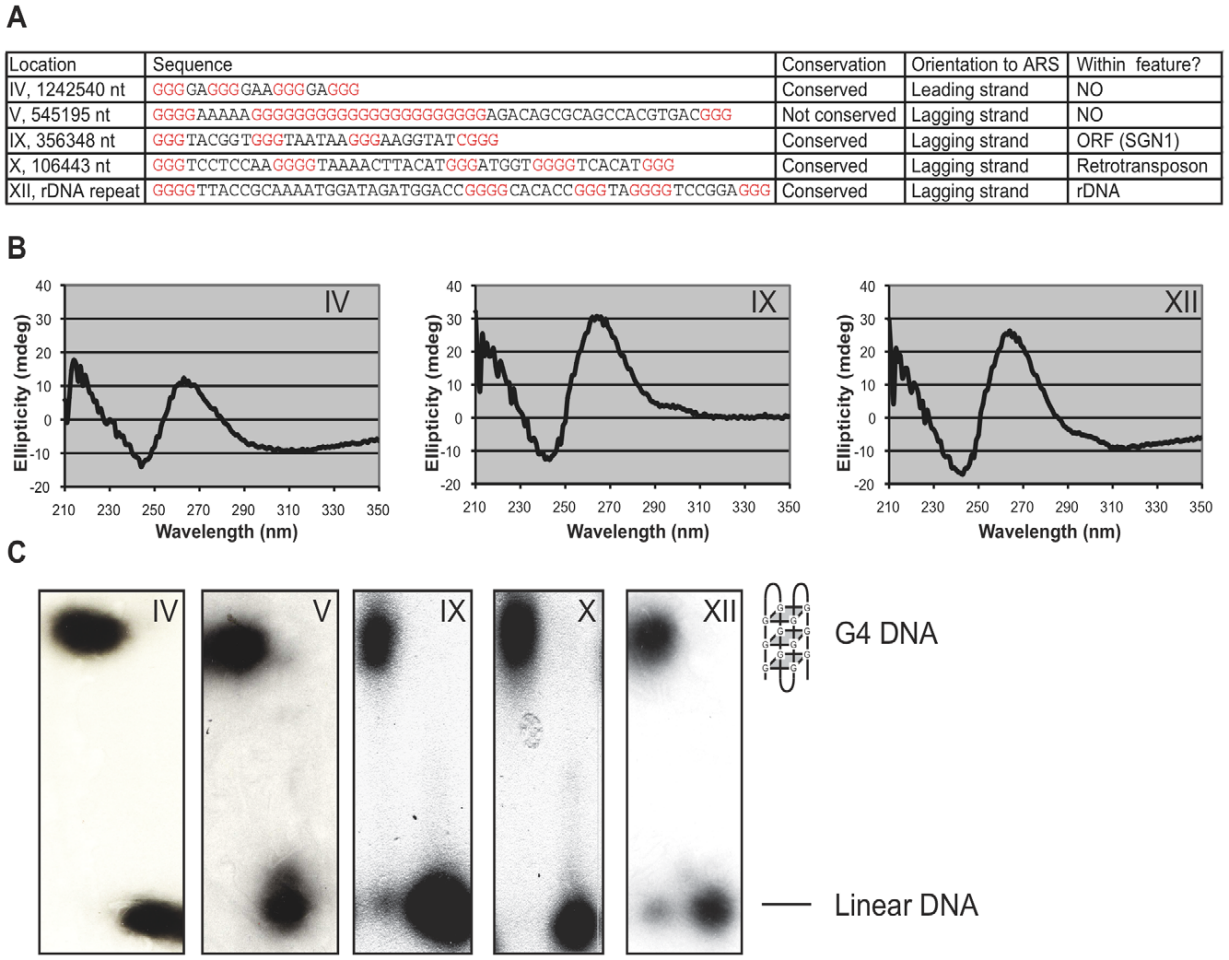
G4 DNA motifs form G4 DNA structures *in vitro*. (**A**) Characterization of the five experimentally tested G4 DNA motifs. The motifs were selected to represent a range of genome locations and conditions. (**B**) Circular dichroism analysis of the three of the five G4 DNA motifs tested demonstrates that they form parallel G4 DNA structures. The G4 motif tested is indicated by the chromosome number above the graph. (**C**) Native acrylamide gel comparing the migration of oligonucleotides before and after the formation of the G4 DNA structure. The G4 DNA structure (indicated by the cartoon on the right) migrates more slowly than linear DNA. The G4 motif tested is indicated by the chromosome number beneath the gel.

We tested the ability of five G4 DNA motifs to form the G4 DNA structure *in vitro* (Fig. 6A). We chose five different G4 DNA motifs, which varied by genomic location, loop length, feature associations, and evolutionary conservation. Since several of the motifs contained relatively long loops, it was not obvious that they would form stable structures. The formation of G4 DNA structures was detected by circular dichroism (CD) spectroscopy, which is a sensitive method for analyzing G4 DNA structures that can discriminate a structure's topology (parallel vs. anti-parallel) [3], [37], and native polyacrylamide gels. Oligodeoxynucleotides containing the predicted G4 DNA motifs were incubated in 1 M NaCl and incubated at 60°C for 48h as previously described [38]. All three of the G4 DNA motifs tested with CD spectroscopy showed clear G4 DNA structure formation at room temperature. Each of the structures formed in a parallel conformation (Fig. 6B). G4 DNA structure formation was also demonstrated for each of the five candidate sequences by the slower mobility of the oligodeoxynucleotides after the formation of the G4 DNA structure in native polyacrylamide gels relative to the mobility of the linear form of the oligodeoxynucleotide (Fig. 6C).

## Discussion

The complementary computational and experimental analyses presented here address the evolutionary conservation and potential function of G4 DNA motifs throughout the *S. cerevisiae* genome. We identified G4 DNA motifs across seven yeast species and found that the motifs are significantly more conserved than expected and are associated with specific functional features of the *S. cerevisiae* genome.

### Evolutionary conservation of G4 DNA motifs

The evolutionary conservation of a G4 DNA motif provides insight into the probability of its formation and function *in vivo*. We determined the conservation in location and sequence of G4 DNA motifs in the *S. cerevisiae* genome among six other fungal species. A significant number of G4 DNA motifs were conserved in location between *S. cerevisiae* and all but the most distantly related fungal genome considered (Fig. 2). Of the over 500 non-telomeric, nuclear G4 DNA motifs, 64% were conserved in at least one other fungal genome, and 34 motifs (7%) were conserved across the *sensu stricto* species.

Positional conservation of G4 DNA motifs does not necessarily imply that the ability to form G4 DNA structures is responsible for the evolutionary constraint. However, the nucleotides in the motif sequences were more conserved than surrounding positions and nucleotides within motifs whose mutation would likely disrupt the ability to form G4 DNA structures were significantly more conserved than other motif nucleotides. This pattern suggests that the observed evolutionary conservation is related to the motif itself rather than to some overlapping feature, and provides substantial evidence for evolutionary pressure to maintain the formation of G4 DNA structures.

Examining the conservation of G4 DNA motifs across six species at a range of evolutionary distances from *S. cerevisiae* (Fig. 2) allowed us to compare the degree of conservation of G4 DNA motifs relative to other genomic features. When analyzing the conservation of ORFs and potential regulatory sequences, considering distant species is often necessary to highlight functionally relevant conservation [21]. G4 DNA motifs are less conserved than ORFs and appear to have qualitatively similar levels of conservation with transcription factor binding sites. It was previously shown that 13% of the representative *S. cerervisiae* Gal4 transcription factor binding sites were conserved across the *sensu stricto* species [22]. In contrast, 7% of G4 DNA motifs were conserved across this evolutionary distance, and very few were conserved beyond the *sensu stricto* species. Lack of conservation does not necessarily imply lack of function; G4 DNA motifs that are not evolutionarily conserved could still form functionally important G4 DNA structures *in vivo*. The presence or absence of G4 DNA structures could generate species-specific differences in functions affected by the structure, such as transcription, replication, or recombination. It is also possible that our definition of conservation, which requires overlapping motifs in sequence alignments, missed patterns in which the specific location of the motif is not as important as its general location relative to other genomic features. For example, the presence of a G4 DNA motif anywhere upstream of an ORF could be more relevant than its specific position within the upstream region.

### Potential functions for G4 DNA motifs suggested by our analysis

We investigated potential functions of G4 DNA motifs by searching for significant associations between the genomic location of the motifs and more than 40 genome features (Table 4 and Methods). Some of the associations we found, including those with promoters and rDNA, were maintained when we considered only evolutionarily conserved G4 DNA motifs (Table 4). The associations with other features such as DSB sites, recombination hotspots, and dubious ORFs, were not maintained across the *sensu stricto* species. It is possible that frequent changes in the locations of these features could explain their lack of conservation. For example, recombination hotspots may change rapidly between species [39], and dubious ORFs in *S. cerevisiae* may actually be functional ORFs in other species. It is also possible that we could not detect these associations because of the small number of conserved motifs and large number of hypotheses tested. Overall, our findings corroborated the previously identified G4 DNA motif enrichment in promoter regions and the rDNA repeat [10]. The known enrichment of G4 motifs in ORFs was refined to show that it may be driven by regions, such as dubious ORFs, that are not confirmed as protein coding. Furthermore, we discovered a number of new associations, such as the significant association of G4 DNA motifs with TF binding sites and non-essential genes.

Perhaps the most unexpected and novel finding of our analysis was the strong association of G4 DNA motifs with sites of frequent DSBs in both unperturbed mitotic and meiotic cells. As there is a significant overlap of preferred DSB sites in mitosis and meiosis (q<0.001), it is possible that G4 structures have a common function in both cell types. The presence of G4 DNA structures at preferred DSB sites suggests two non-exclusive possible functions. First, the formation of G4 DNA structures during DNA replication could increase the probability of DSBs. The formation of these structures during replication when DNA is transiently single stranded could slow or even stall fork progression, leading to DSBs. From yeast to humans, telomeric DNA impedes fork progression during standard semi-conservative DNA replication [40]–[42]. In mammals, this effect is suggested to be due to formation of G4 DNA structures. In addition, mutations in FANCJ, a DNA helicase that unwinds G4 structures *in vitro* [43]–[45], and whose mutation is implicated in a number of human diseases, results in chromosomal deletions in regions with G4 DNA motifs (for review see [46], [47]).

Alternatively, G4 DNA motifs may be associated with DSBs because they have a role in DSB processing. One of the initial steps in DSB repair is processing of the break to generate 3′ single-stranded tails. Formation of a G4 DNA structure in these tails could recruit the DNA damage machinery and influence downstream events. Indeed, several *S. cerevisiae* repair and recombination proteins have activities that affect G4 DNA structures. The Mre11 nuclease, which is one of the first proteins recruited to DSBs, binds and cleaves G4 DNA structures [48]. The Sgs1 DNA helicase, which has important roles in repair and recombination, unwinds G4 DNA *in vitro* [49], and Hop1, a protein that is critical for the synapsis of homologous chromosomes during meiosis, binds and promotes the formation of G4 DNA structures *in vitro* [50], [51]. Meiotic DSBs are created by the topoisomerase-like protein Spo11 [35], [52], but no DNA sequence preference for Spo11 action has been identified. If Spo11 acts at G4 DNA structures, this preference may have been missed by motif finding programs due to the varying size and sequence of loops in G4 DNA motifs.

Another important and unexpected finding from our analysis is the high density of G4 motifs in the AT-rich mtDNA (Fig. 5). Due to the lack of mtDNA sequence data for most of the species used for our conservation analysis, we could not assess the evolutionary conservation of the mitochondrial G4 DNA motifs. The occurrence of these motifs with respect to annotated genome features was very different from what was observed for nuclear DNA, as only two out of the 32 motifs overlapped a feature (q<0.001). mtDNA exhibits a high frequency of spontaneous mutation and recombination [53]. G4 DNA motifs in the mtDNA may influence recombination or repair within the mtDNA, because formation of the G4 DNA structure could lead to DSBs (as discussed above for nuclear DNA). In particular, the distribution of mitochondrial G4 DNA motifs suggests that they could help limit recombination in the mtDNA to non-genic regions.

Several helicases, including human BLM, WRN, and FANCJ, that function throughout the genome, can unwind G4 DNA structures. Mutation of the human versions of these helicases causes inherited diseases characterized by genome instability and increased cancer risk [44], [49], [54], [55]. It is intriguing that the *S. cerevisiae* Pif1 helicase, which also unwinds G4 DNA structures *in vitro* [54], affects the maintenance and replication of telomeres [56], rDNA [40], and mtDNA [57]—three DNA regions with particularly high densities of G4 DNA motifs. A role for the Pif1 helicase in promoting genome stability by acting at G4 DNA structures is supported by our recent finding that Pif1 is preferentially associated with both G4 DNA motifs and preferred sites of mitotic DSBs *in vivo* (KP, JAC, and VAZ, in preparation).

### Conclusion

Yeast provides an ideal system in which to study predicted G4 DNA motifs *in vivo*. Now that potential sites of G4 DNA structures have been identified in yeast and mapped relative to genomic features, they can be mutated to determine their contribution to specific functions, such as transcription, replication fork movement, and DSB formation. The conserved *S. cerevisiae* G4 DNA motifs identified here are especially attractive for experimental characterization. In addition, the computational techniques applied to locate and analyze these motifs are general, and could easily be used in comparative evolutionary analyses of G4 DNA motifs in other organisms, such as flies and human.

## Methods

### Sources of genomic data

The sequences for all chromosomes in the *S. cerevisiae* S288C genome were downloaded from the SGD on October 17, 2008. These were used in the analysis of the distribution of G4 DNA motifs and in most of the association tests.

For analysis of the conservation of G4 DNA motifs and nucleotides, multiple sequence alignments of the Oct. 2003 assembly (sacCer1) of the *S. cerevisiae* genome with the sequences of *S. paradoxus*, *S. mikatae*, *S. kudriavzevii*, *S. bayanus*, *S. castelli*, and *S. kluyveri* were downloaded from the UCSC Genome Browser. In short, they used blastz and multiz to create the alignments, and phastCons to compute the conservation scores for all aligned nucleotides in *S. cerevisiae*. Though there are slight differences in the two drafts of the *S. cerevisiae* genome considered here, all G4 DNA motifs found in the most recent version are present in the Oct. 2003 version.

The genome annotations used in the region association analysis come from a variety of sources. Genome features as of March 7, 2009 were downloaded from SGD. These include: ARS_consensus_sequence, ARS, CDEIII, CDEII, CDEI, CDS, centromere, dubious-ORF, five_prime_UTR_intron, gene_cassette, intron, long_terminal_repeat, mating_locus, ncRNA, noncoding_exon, ORF, promoter1000, pseudogene, repeat_region, retrotransposon, rRNA, snoRNA, snRNA, telomere, transposable_element_gene, tRNA. Regulatory features as defined by the SGD were downloaded on March 9, 2009. Regions 100 and 1000 nt upstream of ORFs were also considered. In addition to these features, essential genes were taken from [58]; origins of replication came from [59]. The set of highly transcribed genes came from [60], [61], and the list of upstream ORFs (uORFs) was taken from [61]. Initial association results for γ-H2AX binding sites were based on a data set generated using an Affymetrix high-density genome-wide array and generously provided by M. Grunstein (personal communication). We confirmed these findings on γ-H2AX binding sites as determined by ChIP on chip using a genome-wide array from Agilent (G4493A). Double strand break sites were taken from [35]. Single and double promoters come from [62].

### Identification and filtering of G4 DNA motifs

We performed a regular expression search on the DNA sequence of each *S. cerevisiae* chromosome to find sequence patterns with the potential to form G4 DNA structures. All instances of four or more G-tracts separated from one another by short loop regions were identified. Loop length thesholds between 5 and 50 nt were considered, but unless otherwise stated the results in the paper are based on a 25 nt loop length limit. Sequence regions that contained more than four G-tracts separated by loops were combined into a single motif; these sequences have the potential to form one of several topologically distinct G4 DNA structures. Source code for the search program is available from the authors by request. The genomic location and sequence of all motifs is available as Dataset S1.

In previous studies, two different constraints have been placed on the G4 DNA motif pattern when searching for matching sequences. The most common approach has been to limit the length of the loop regions between G-tracts [11]–[13], [25]. Other authors have placed a limit on the overall length of the motif [10], [14]. We choose to use the more commonly used loop length threshold approach, because only constraining motif length can result in relatively short motifs with one long loop and two very short loops. However, there is a large degree of overlap between the motifs defined by these two approaches, and our feature association tests produced very similar results on motif length limited sets (data not shown).

Telomeric DNA in most organisms, including *S. cerevisiae*, consists of repetitive G-rich DNA sequence that is conducive to forming the G4 DNA structure [5], [63]. Our search identified 80 G4 DNA motifs in the ∼141 kb of subtelomeric and telomeric sequence identified in SGD. However, due to the difficulty of sequencing and processing telomeric regions, they are under-represented in the *S. cerevisiae* genome sequence. As a result, the conservation of telomeric G4 DNA motifs is difficult to quantify. All analyses in this paper are limited to non-telomeric G4 DNA motifs. Similarly, the sequence of chromosome XII in the SGD datababase contains only two copies of the rDNA (ribosomal DNA) repeat [64], while cells contain ∼150 rDNA repeats. We consider only one rDNA repeat, and the G4 DNA motifs found therein, in our analysis. After these filtering steps, 552 of the 668 initial G4 DNA motifs remained.

Conservation analysis could be performed only if the sequence of interest could be aligned to a homologous region in another species. Therefore, we limited our analysis to the 507 of the 552 non-telomeric, non-mitochondrial *S. cerevisiae* G4 DNA motifs found in genomic regions that were aligned to a corresponding DNA region in at least one of the six other species in the multiz alignments from the UCSC Genome Browser [65]. (The conservation of mitochondrial G4 DNA motifs could not be analyzed, because the sequences of the mitochondrial genomes are not available for many of the species considered here.)

### Conservation of G4 DNA motifs across species at the motif level

The alignments obtained from the UCSC Genome Browser consist of blocks of sequence from the *S. cerevisiae* genome sequence that can be aligned to one or more other yeast species. For each G4 DNA motif in *S. cerevisiae* that is in such a region, we scanned the aligned sequence from the other species for the G4 DNA motif pattern. If a motif was found that overlapped the *S. cerevisiae* motif, then the motif was said to be conserved in the current species. The conserved motif was not required to be identical to the overlapping *S. cerevisiae* motif.

Once the conservation of each G4 motif was determined, we computed whether or not the number of motifs conserved between *S. cerevisiae* and each species was greater than would be expected by chance. We obtained an empirical p-value for the observed number of conserved motifs by “evolving” each aligned *S. cerevisiae* sequence block according to a local nucleotide transition matrix that reflected the observed probabilities of nucleotide change between the pair of aligned sequences. We generated 10,000 such evolved sequences with the same length, substitution probabilities, and evolutionary distance, and scanned each for a G4 motif that overlaps the *S. cerevisiae* motif. These steps provided the background distribution from which we calculated an empirical p-value for the observed number of conserved G4 motifs between each pair of species. We performed the same test, using the global nucleotide transition matrix determined by considering all alignments between the two species, and the results were similar, but with smaller p-values.

### Conservation of G4 DNA motifs across species at the nucleotide level

For each nucleotide position in the alignments containing *S. cerevisiae* G4 DNA motifs, we considered conservation scores calculated by the phastCons package [27]. We compared the average conservation scores of positions within the G4 DNA motif to those in an immediate neighborhood of 100 bases on either side of the motif. We also compared the conservation of disruptive and non-disruptive positions within the G-tracts [16]. The statistical significance of the difference between these sets of conservation values was computed using the Wilcoxon rank sum (Mann-Whitney U) test as implemented in R [66].

### G4 DNA motif associations with genomic regions

To investigate potential functional roles of the G4 DNA structure, we analyzed the association of G4 DNA motifs with other known genome features. Given a query set of genome regions (e.g., G4 DNA motifs) and a reference set (e.g., all promoter regions), the number of overlaps between the reference and query sets was determined. For most features we considered the strand on which it occurred when determining associations. However, this information was not available or relevant for all features. We also considered associations of the G4 DNA motifs with expanded forms of some genomic features, e.g., with a window of 500 nucleotides on either side of a short feature such as an Autonomously Replicating Sequence (ARS). If a window was used in determining a given association, it is reported in the text.

The number of overlaps between two sets of genome regions expected by chance depends on their length and distribution across the chromosomes. To evaluate the significance of the observed number of overlaps between two sets of genome regions, we generated 1,000 random region sets that matched the number and length distribution of the query set at the chromosome level. That is, if the query set consisted of three G4 DNA motifs on chromosome I, then each of the 1,000 random query region sets had three randomly placed regions on chromosome I of the corresponding lengths. For each set of randomly placed query regions, we calculated the number of overlaps with the reference region set. We then compared the number of observed overlaps to the random distribution of overlaps to obtain an empirical p-value for the association of the regions. The false discovery rate (FDR) control as implemented in the R q-value package was used to produce q-values that account for the multiple comparisons.

### Genome wide γ-H2AX analysis

For the genome wide γ-H2AX analysis, DNA was chromatin immunoprecipitated using anti-H2A phosphoS129 (Abcam polyclonal Ab #ab15083). DNA was amplified and labeled according to the instructions described in the Agilent Yeast ChIP on chip analysis protocol v9.2 (http://www.chem.agilent.com). Briefly, after PCR amplification of ChIP DNA, DNA was labeled and purified according to the manufactures handbook using Cy3- or Cy5-coupled dUTP using the BioPrime Array CGH Genomic Labeling Module (Invitrogen, http://www.invitrogen.com, Cat No 18095-011). For our genome wide association we used the microarrays from Agilent Technologies (Cat No G4486A).

Labeled DNA (2.5 µg of Cy3 labeled input DNA and Cy5 labeled ChIP DNA) together with 10× Agilent Blocking Agent and 2× Agilent Hybridization buffer, were prepared for hybridization. Arrays were hybridized at 65°C for 18 h at 20 rpm. After hybridization, the array slides were washed according to the Agilent SSPE Wash protocol and scanned with an Agilent Microarray Scanner. Data extraction, including dye normalization and spatial detrending, was done using the default settings of the Agilent Feature Extractor. The resulting log_2_ ratio of Cy3 and Cy5 signal in each feature was used to determine the location of chromatin immunoprecipitaed DNA. Genome wide γ-H2AX analysis was repeated three times, and peaks were defined using a p-value threshold of 10^−4^. The γ-H2AX binding sites are listed in Dataset S5.

### Formation and analysis of G quadruplex DNA structures

Oligodeoxynucleotides were synthesized by IDT (USA). The concentrations of all oligodeoxynucleotides were estimated using extinction coefficients provided by the manufacturer. All sequences tested are listed in Fig. 6A. Formation of the G4 DNA structure was performed as described in [38].

G4 DNA structure formation was confirmed by non-denaturing polyacrylamide gel electrophoresis (PAGE). Before and after the formation of the G4 DNA structure, oligodeoxynucleotides were 5′ labeled with T4 polynucleotide kinase (NEB) and γ^32^P-ATP, resolved by 12% non-denaturing PAGE, and visualized using phosphoimaging.

Circular dichroism (CD) spectroscopy was recorded on a 62DS AVIV spectropolarimeter using a 1 cm path length quartz cuvette in a reaction volume of 900 µl. Scans were performed at 25°C over a wavelength range of 210–350 nm with a scanning speed of 500 nm/min, a response time of 1 s, 1 nm data pitch and 1 nm bandwidth.

## Supporting Information

Dataset S1 Genome locations of G4 DNA motifs.
(0.03 MB TXT)

Dataset S2 Genome locations of conserved G4 DNA motifs
(0.00 MB TXT)

Dataset S3 Association statistics for *S. cerevisiae* non-mitochondrial, non-telomeric G4 DNA motifs with genome features.
(0.01 MB TXT)

Dataset S4 Association statistics for conserved *S. cerevisiae* G4 DNA motifs with genome features.
(0.01 MB TXT)

Dataset S5 Genome-wide γ-H2AX binding sites.
(0.32 MB CSV)
